# Complement as driver of systemic inflammation and organ failure in trauma, burn, and sepsis

**DOI:** 10.1007/s00281-021-00872-x

**Published:** 2021-06-30

**Authors:** Marco Mannes, Christoph Q. Schmidt, Bo Nilsson, Kristina N. Ekdahl, Markus Huber-Lang

**Affiliations:** 1 Institute of Clinical and Experimental Trauma-Immunology, University Hospital of Ulm, Helmholtzstr. 8/2, 89081 Ulm, Germany; 2 Institute of Pharmacology of Natural Products and Clinical Pharmacology, Ulm University, Ulm, Germany; 3 Department of Immunology, Genetics and Pathology (IGP), Rudbeck Laboratory C5:3, Uppsala University, Uppsala, Sweden; 4 Linnaeus Center of Biomaterials Chemistry, Linnaeus University, Kalmar, Sweden

**Keywords:** Trauma, Burn, Sepsis, Complement activation, Thromboinflammation, Systemic inflammation, Clinical translation

## Abstract

Complement is one of the most ancient defense systems. It gets strongly activated immediately after acute injuries like trauma, burn, or sepsis and helps to initiate regeneration. However, uncontrolled complement activation contributes to disease progression instead of supporting healing. Such effects are perceptible not only at the site of injury but also systemically, leading to systemic activation of other intravascular cascade systems eventually causing dysfunction of several vital organs. Understanding the complement pathomechanism and its interplay with other systems is a strict requirement for exploring novel therapeutic intervention routes. Ex vivo models exploring the cross-talk with other systems are rather limited, which complicates the determination of the exact pathophysiological roles that complement has in trauma, burn, and sepsis. Literature reporting on these three conditions is often controversial regarding the importance, distribution, and temporal occurrence of complement activation products further hampering the deduction of defined pathophysiological pathways driven by complement. Nevertheless, many in vitro experiments and animal models have shown beneficial effects of complement inhibition at different levels of the cascade. In the future, not only inhibition but also a complement reconstitution therapy should be considered in prospective studies to expedite how meaningful complement-targeted interventions need to be tailored to prevent complement augmented multi-organ failure after trauma, burn, and sepsis.

This review summarizes clinically relevant studies investigating the role of complement in the acute diseases trauma, burn, and sepsis with important implications for clinical translation.

## Introduction

Medical care has been extraordinarily improved for a variety of diseases in the last decades. This is because of the increasing knowledge on the pathophysiology but also due to growing awareness about risk factors fostering the development of preventive arrangements that reduce the disease burden. However, for acute diseases including trauma and burn which can hit an individual independently of gender and age, preventive medical intervention is not possible. This is illustrated by the fact that trauma is a major cause for morbidity and mortality within the younger population [1]. Globally, almost 5 million people died as a consequence of traumatic injuries in 2013 [2], and approximately 120,000 deaths were registered to be associated with heat and hot substance injuries in 2017 [3]. If initial injuries are not fatal, patients have to deal with a complex immune reaction characterized by a strong activation of the complement system and a massive release of pro-inflammatory molecules that aim to aid healing but instead can turn into induction of life-threatening inflammation [4]. Disruption of the physical skin barrier caused by trauma or burn facilitates infiltration of bacteria, thus increasing the susceptibility to sepsis. Especially burn injuries are described to be associated with a very high risk of sepsis [5]. Since the complement system is a major disease driver in the acute phase after injury, it is of paramount importance to understand its specific role in traumatic scenarios and integrate those into the body’s overall immune surveillance.

This review aims to summarize the role of the complement system in the settings of trauma, burn, and sepsis with a focus on the clinical and therapeutical aspects.

## As old as trauma, burn, and sepsis: the complement system

The complement system functions as an alarm system and accounts for a substantial part of the innate immunity fighting at the sharp end against invaders. However, having initially believed to be just a heat-labile factor that “complements” the action of antibodies by forming complexes with antimicrobial properties [6], in the last decades, the complement system was found to participate in a wide range of physiological and pathophysiological conditions. Inflammatory disturbance, organ development, thrombotic disorders, and tissue regeneration have been established as processes with crucial contributions by different complement activation products [7–10]. Uncontrolled complement activation and imbalanced regulation caused by injuries like trauma, burn, and sepsis can deteriorate the outcome of patients.

Complement is classically a “three-arm” branched system acting in a cascade-like fashion. It consists of more than 50 proteins, many of them being zymogens which become activated upon proteolytic cleavage. The activation of the classical pathway (CP) is based on the recognition of IgM or multimeric (ideally hexameric) IgG antibody patterns by C1q which entails a conformational change in C1q eventually leading to the activation of its associated proteases C1r and C1s. The latter of which can cleave C4 and C2, together forming the classical pathway C3 convertase C4b2a. The same convertase, albeit by the action by the MASP-1 and -2 proteases, also emanates from the lectin pathway (LP) when pattern recognition molecules such as mannose-binding lectin, ficolins, and collectins perceive microbe-associated molecular patterns (MAMPs). In addition, many of these recognition molecules (including C1q) also identify various damage-associated molecular patterns (DAMPs), i.e., altered self-structures which are released by or exposed on necrotic and/or apoptotic cells and bodies, thereby initiating complement activation.

While the classical and lectin pathways are turned on specifically, the alternative pathway (AP) is active all the time. At a low level, C3 activates continuously and indiscriminately assembling early AP convertases that cleave C3 into C3b [11]. All pathways converge at the proteolytic cleavage of the central protein C3 generating the anaphylatoxin C3a and the opsonin C3b. The latter exposes a hidden thioester moiety allowing the covalent attachment especially to free hydroxyl or amino groups. Released anaphylatoxins attract immune cells to the site of infection. By binding to its receptor (C3aR), C3a elicits cellular responses (e.g., release of histamines from mast cells [12]). Of note, the role of C3a is not consistently pro-inflammatory as a reevaluation emphasized (reviewed in [13]). Nevertheless, C3a plasma concentrations directly correlate with the severity of trauma and adumbrate the development of multi-organ dysfunctions [14].

If not stopped by the action of complement regulators (as it happens on healthy host surfaces), any initially produced C3b amplifies via the AP (auto-)amplification loop: membrane-bound C3b serves as basis for the formation of further AP convertases which generate more C3b molecules, thereby amplifying the C3 deposition exponentially. Increasing C3b densities deposited on surfaces bind and prime C5, thus preparing C5 to be cleaved by bimolecular convertase complexes, a crucial step for the onset of the terminal complement pathway [15–17]. Conversion of C5 leads to the release of the highly potent anaphylatoxin C5a and C5b, the initiating molecule of the membrane attack complex (MAC). Binding of C5b to the late complement proteins C6, C7, C8, and multiple copies of C9 can lead to integration of this complex into the cell membrane causing lysis due to osmotic flux [18]. When assembling of MAC is quenched by S protein (vitronectin) before insertion into a bilayer membrane, a soluble complex-denominated sC5b-9 is formed [19]. In contrast to C3a, two C5a receptors (C5aR1 and C5aR2/C5L2) are known. The role of C5aR2 is divergent as pro-inflammatory and anti-inflammatory properties are reported for C5aR2 [20, 21]. Under conditions of strong complement dysregulation, host cells can be affected by MAC possibly inducing apoptosis among other effects. While erythrocytes have been shown to be especially susceptible for MAC lysis [22], nucleated cells appear more resilient. Tutelar mechanisms of nucleated host cells include, for instance, shedding of MACs via microvesicle formation, thus reducing the harmful burden [23]. Next to cytolysis, also sub-lytic MAC concentrations have been shown to trigger important immunomodulatory functions, such as the release of the inflammatory mediators IL-8 and MCP-1 or the upregulation of the adhesion molecule P-selectin which was demonstrated for human umbilical vein endothelial cells [24–26].

## Complement activation requires strict control mechanisms: the regulators

These few examples illustrate the enormous potential of the complement effector functions and forebode their tight regulation. The assembly of lytic MAC pores can be intercepted by CD59 (protectin), a membrane-anchored complement regulator which disturbs the incorporation and inhibits the polymerization of the pore-forming C9 molecules. Interestingly, cell damage can trigger shedding of soluble CD59 (sCD59), evidenced by elevated plasma levels which were detected after acute myocardial infarction [27]. The concrete function of sCD59 as well as the degree to which its plasma concentration could be a useful biomarker in other clinical settings (e.g., trauma) has to be examined in prospective studies. Beside CD59, host cells are equipped with different other membrane-bound complement regulators. Since the convertase-mediated activation of the central proteins C3 and C5 is a crucial step in the cascade, convertases require tight regulation beyond the intrinsic convertase decay which occurs within minutes and disallows reformation of its components once separated. If their formation takes place on body’s own surfaces, convertases are dissociated by the action of the decay accelerating factor (DAF, CD55), complement receptor 1 (CR1, CD35), or soluble regulators bound to the host surface like factor H (FH; specific for the AP) or the C4b-binding protein (C4BP, specific for the CP/LP) [28, 29]. Consequently, C3 deposition and further downstream effector functions can be blocked which was previously shown, e.g., in vitro [30] and in vivo by the administration of recombinant DAF to pigs subjected to hemorrhagic shock. Decreased C3 deposition and organ damage correlated positively with animal survival in this model [31]. In another model in rats, early application of recombinant DAF after traumatic chest injury resulted in beneficial outcome regarding cytokine profile confirming a possible therapeutic role for recombinant DAF although the exact mode of action remains unclear and is still content of research [30, 32]. Next to DAF, also CR1 decays convertases, completing the group of known membrane-bound molecules exhibiting decay accelerating activity. CR1 also harbors co-factor activity for factor I (FI)–mediated cleavage of C4b and C3b into iC4b and iC3b and further breakdown of products. Such inactivated opsonins lose their ability to form convertases but still function as ligands for a variety of complement receptors expressed on a wide range of immune cells. A putative therapeutical benefit of CR1 was demonstrated by the application of soluble CR1 (sCR1) into rats protecting nerves from early axon loss after crush injury [33]. The membrane co-factor protein (MCP, CD46) completes the “classical” surface-bound regulators. It serves as a co-factor for the FI-mediated inactivation of C3b and C4b [34]. Interestingly, declined MCP levels on neutrophils have been measured after trauma, implying a lower self-protection profile in this setting [35]. In sepsis, MCP participates in expediting the immune response by modulating the polarization and survival of macrophages [36]. Of note, beside its primary complement-related functions, MCP was described as a “pathogen magnet” [37]. Several viral as well as bacterial species were identified to employ MCP as a docking receptor to enter cells, among others the pathogenic bacterium *Streptococcus pyogenes* [38].

In addition to the described membrane-bound regulators, complement also comprises soluble regulators which stop inadequate complement consumption in the fluid phase and reinforce surface regulation by the membrane-fixed regulators. C4BP, the fluid phase regulator of the CP, binds C4b and facilitates its degradation by FI and also decays the classical C3 convertase C4b2a [39, 40]. Interestingly, some pathogens specifically capture C4BP, thus probably evading the actions of the immune system [41]. C4BP has been shown to inhibit protein S (not to be confused with “S protein”, see above), an important co-factor for the protein C–mediated downregulation of the coagulation cascade [41]. By contrast, antithrombotic properties have been postulated for the most upstream soluble C1 inhibitor (C1INH) which controls the activation of C1. Application of C1INH in a murine model of traumatic brain injury displayed anti-inflammatory effects as well as reduced fibrinogen deposition and diminished vessel occlusion in comparison to sham-treated mice [42]. Interestingly, also an (complement) inactive form of C1INH has been demonstrated to protect from sepsis in a cecal ligation and puncture (CLP) mouse model [43]. The only known negative AP regulators in the fluid phase are FH and its splice variant factor H-like 1 (FHL-1). Both molecules share most of their functions which are co-factor activity for FI-mediated cleavage of C3b and decay accelerating activity for C3bBb convertases. Since FH has improved host recognition properties in comparison with FHL-1 [44], the physiological need of FHL-1 is not completely clear so far and content of ongoing research [45]. In addition to FHL-1, the FH family also comprise several factor H–related proteins (FHRs) which share ligands with FH [46, 47]. FHRs are expected to further fine-regulate the AP regulation by FH, but the precise roles are still under investigation. One recent study shows that FHR-1 and FHR-5 interact with DNA and necrotic cells, thereby displacing FH and increasing opsonization and complement activation [48].

Taken together, the complement system is stringently controlled at different levels of the cascade, allowing attack when necessary and suppressing it when not appropriate. However, acute diseases like trauma, burn, or sepsis unbalance the cascade by triggering an overwhelming complement activation response initiated by the enormous abundance of DAMPs and/or MAMPs. This unbalanced response is further influenced by the altered expression level of specific plasma proteins (e.g., C-reactive protein (CRP), serum amyloid A, fibrinogen, haptoglobin) in the course of the acute phase response [49]. Among them, differences in the expression levels of central complement proteins have been reported as well: C4 and concomitantly CRP serum concentrations were significantly higher in patients with severe acute respiratory syndrome in comparison to the control group [50], and C3 and CRP levels correlated in subjects with unstable angina [51]. In turn, CRP can activate the classical pathway potentially increasing the complement dysregulation in acute diseases. One study could reveal that complement is even a driver of the general process of the acute phase response since human CRP transgenic mice deficient in C3 or C5 have been shown to attenuate LPS-induced upregulation of the CRP gene [52]. As the complement cascade also cross-talks with other physiological pathways like hemostasis [53, 54], severe injuries do not only lead to dysregulation of the complement response but also offset the balance in other important effector systems.

## Thromboinflammation

The intravascular innate immune system (IIIS) consists of the blood cascade system, namely the coagulation system, the kallikrein/bradykinin system (contact system), and the fibrinolytic system, in addition to the complement system which has been described in detail above. The IIIS is the humoral arm of the natural immune system, and 95% of all animal species alive today rely on this line of defense which functions without involvement of T and B cells. A key function of the IIIS is to distinguish between self (healthy autologous cells), non-self (MAMPs), and/or altered self (DAMPs), but unlike the adaptive immune system, the IIIS can only differentiate itself from non-self at the species level and not at the individual level.

There are multiple points of contact and cross-talk between the systems within the IIIS, e.g., when a proteolytic enzyme which has been activated in one system can activate a substrate molecule within another system [55]. One example of high relevance for this article is the cleavage of complement components C3 and C5 to yield functional anaphylatoxins by non-canonical proteases. These include factors IXa, Xa, and thrombin as well as plasmin which are unleashed during activation of the coagulation and fibrinolytic system, respectively [56–58]. Cross-talk which occurs in the opposite direction is exemplified by MASP-1 and -2 which play significant roles in coagulation activity and can lead to fibrin formation in the absence of thrombin [59, 60].

However, research in this area has up to recently been very segregated, mainly for technical reasons. Consequently, many of the potential interactions have not been demonstrated, and altogether, their physiological relevance in different pathological settings is not always clear. The main caveat is that researchers in the various disciplines traditionally tend to use different anticoagulants according to the requirement of one system, but which may inactivate another system, thereby disabling studies of their interactions. Typically, complement activation is studied in serum where all coagulation activation is irreversibly inhibited. On the other hand, coagulation is monitored in citrate plasma because its activation can be restored by recalcification, but complement is activated and consumed under these conditions. This has led us and other colleagues to do studies in whole blood, preferably completely without soluble anticoagulants to enable mapping of interaction between various cascade systems and their target cells [61, 62].

The cascade system of blood is etiologically ancient and has an important function as a “waste disposal system” where recognition and removal of foreign substances and particles, including apoptotic and necrotic cells, are central. The proteins in the various cascades have a common origin, and parent molecules can often be identified in evolutionary early organisms such as sea urchins. The systems have been developed through gene duplications either through the two genome duplications that have taken place during evolution or through gene duplications taking place locally, which creates clusters of genes coding for proteins with similar functions. One poignant example is C3 [63, 64]: a C3-like molecule is found early in evolution, and after two gene duplications, four homologous proteins are now found in vertebrates, i.e., C3, C4, C5, and also the proteinase inhibitor α2M (with a similar mechanism of action). Local duplications have led to the cluster of genes for complement inhibitors found on chromosome 1 that encode FH, DAF, MCP, C4BP, etc. [65]. Similar duplications have taken place within other cascade systems, which is one explanation of the complexity of these cascade systems.

Activation of thromboinflammation is a driving force in several diseases and pathological conditions including trauma, burn injuries, and sepsis which are the main focus of this article and discussed in detail. Other clinically relevant conditions include ischemia-reperfusion injury (IRI) which may be triggered by thrombotic events such as cardiac infarction and stroke but also by therapeutic interventions like transplantation and cardiopulmonary bypass. The concept of thromboinflammation and various pathophysiological implications is discussed in detail in Ekdahl et al. [66].

## Trauma

The complex innate immuno-pathophysiological response after trauma has recently been extensively reviewed on a cross-talking multiple organ level [67, 68]. Concerning the basic principles of the complement response after trauma including the development of a temporary complementopathy, we refer to our latest review [24]. Here, we will mainly focus on the role of complement in the clinical setting summarizing meaningful studies in higher species (non-human primates) in addition to clinical therapeutic aspects in man.

Caused by the abovementioned DAMP/MAMP– and coagulation–driven mechanisms, any trauma leads to local complement activation and, if the tissue injury is severe enough, also to systemic activation. In the case of life-threatening polytrauma, rapidly enhanced C3a/C3 ratios as well as C5a and sC5b-9 plasma concentrations in patients have been reported by several groups [69–73]. Based on an excessive complement activation, a drop in complement functional performance as a temporary complement dysfunction (or “traumatized complement”) has been postulated, likely setting the patient at an enhanced risk of infectious complications [24, 71, 72]. On the cellular level, neutrophils have lost their C5aR1 and C5aR2 and altered their complement regulatory proteins after severe tissue injury which has been associated with several neutrophil dysfunctions [35, 74]. In the intensive care unit setting, loss of C5aR1 on neutrophils has even been proposed as a reliable predictive marker for development of subsequent infectious complications [75]. However, it is still a matter of debate whether and to which extent exogenous proteases, e.g., from the coagulation or fibrinolytic cascade, contribute to early complement activation after trauma [58, 76–80]. Here, further research is needed, especially to determine the clinical implications of non-canonical complement activation by tissue trauma.

Excessive complement activation after severe tissue injury and the associated multidimensional immune, cellular, and microvesicle functions provide some rationale to target specific complement factors for more favorable cell, organ, and clinical outcomes [81]. Nevertheless, it has to be emphasized that not only inhibition but also complement reconstitution strategies may be helpful depending on the immune status, the targeted factor, and/or the complement pathway involved. For example, fresh frozen plasma, a major component of early resuscitation intervention in polytrauma patients with hemorrhagic shock and coagulation disorders, contains not only various clotting factors but also multiple complement components, such as C3 and C5. However, the level of active complement in fresh frozen plasma depends on donor, age, and storage factors, making it difficult to correlate clinical improvement with supplement complement functionality via plasma infusions [82]. Moreover, preparations of packed platelets were demonstrated to contain anaphylatoxins, which even increase with storage time [83]. Besides transfusion of blood products, patients often receive tranexamic acid early after polytrauma to treat bleeding disorders by inhibiting plasmin and thus hyperfibrinolysis. This lysine analogue also significantly reduced complement activation as reflected by decreased systemic sC5b-9 (also called terminal complement complex, TCC) concentrations in patients with hemorrhagic shock [84]. Mechanistically, either a direct inhibition of complement activation or an indirect inhibition occurs via blockade of plasmin which in turn loses its C3 or C5 cleaving ability.

Excessively generated complement activation products such as C3a and C5a can be cleared by hemoadsorbance, an intervention which has shown some protective effects in major surgery so far [85]. In contrast to a global reduction of circulating complement cleavage products, a few targeted complement inhibition strategies have been evaluated in humans and in monkeys. C1 inhibition was applied in trauma patients with femur fracture with the primary read-out for changes in IL-6 concentrations reflecting systemic inflammation [86]. However, based on a heterogenous cohort and recruitment challenges, this study has been closed, and no conclusion could be drawn so far. Effective blockade of the central C3 component by Cp40 (a member of the compstatin family) in a delayed manner, i.e., 30 min after induction of a severe trauma/hemorrhagic shock (MAP 30 mmHg for 60 min), significantly reduced the inflammatory response and improved hemodynamics, barrier, and several organ functions [87]. On the C5 level, in a non-human primate hemorrhagic shock/polytrauma model, the authors confirmed the clinical findings of elevated C5 cleavage products and tested ex vivo some small peptide inhibitors for C5 cleavage [88]. However, no in vivo inhibition has been performed so far in such a model. To our knowledge, selective inhibition of MAC formation has not been performed yet. In conclusion, although complement seems a major driver of the posttraumatic inflammatory response, there is a clear lack of clinical studies targeting central complement components or neutralizing the excess of generated complement activation products after trauma and during hemorrhagic shock. It is tempting to speculate that a remaining, albeit low, activity of complement for opsonization of pathogens and clearance of infected or damaged cells is desirable in the context of trauma to prevent viral reactivation and infectious complications and to aid the reconstitution of tissue homeostasis [89].

## Burn

Burn as physical injury is the fourth most common trauma worldwide [90]. Primarily, the term “burn” is associated with fire and injuries caused by hot substances. Beyond that, radioactive and electric injuries as well as caustic chemicals may also elicit burn-like wounds. Even cold wounds have been shown to release comparable cytokine profiles although there are important morphological differences in comparison to heat wounds [91]. Evaluation of the burn dimension plays a crucial role in the acute medical care. On the one hand, burn severity is categorized depending on the wound depth ranging from an epidermal first-degree over a second- and third-degree to a fourth-degree full-thickness burn [92]. On the other hand, an important parameter is the affected total body surface area (TBSA) which has been shown to directly correlate with morbidity and mortality. Patients experiencing 60% TBSA or more displayed increasing hyper-inflammatory reactions as well as a drastic diminished cardiac function [93]. Such hyper-inflammatory responses are predestinated to show a strong activation of the complement system.

**Figure 1.**
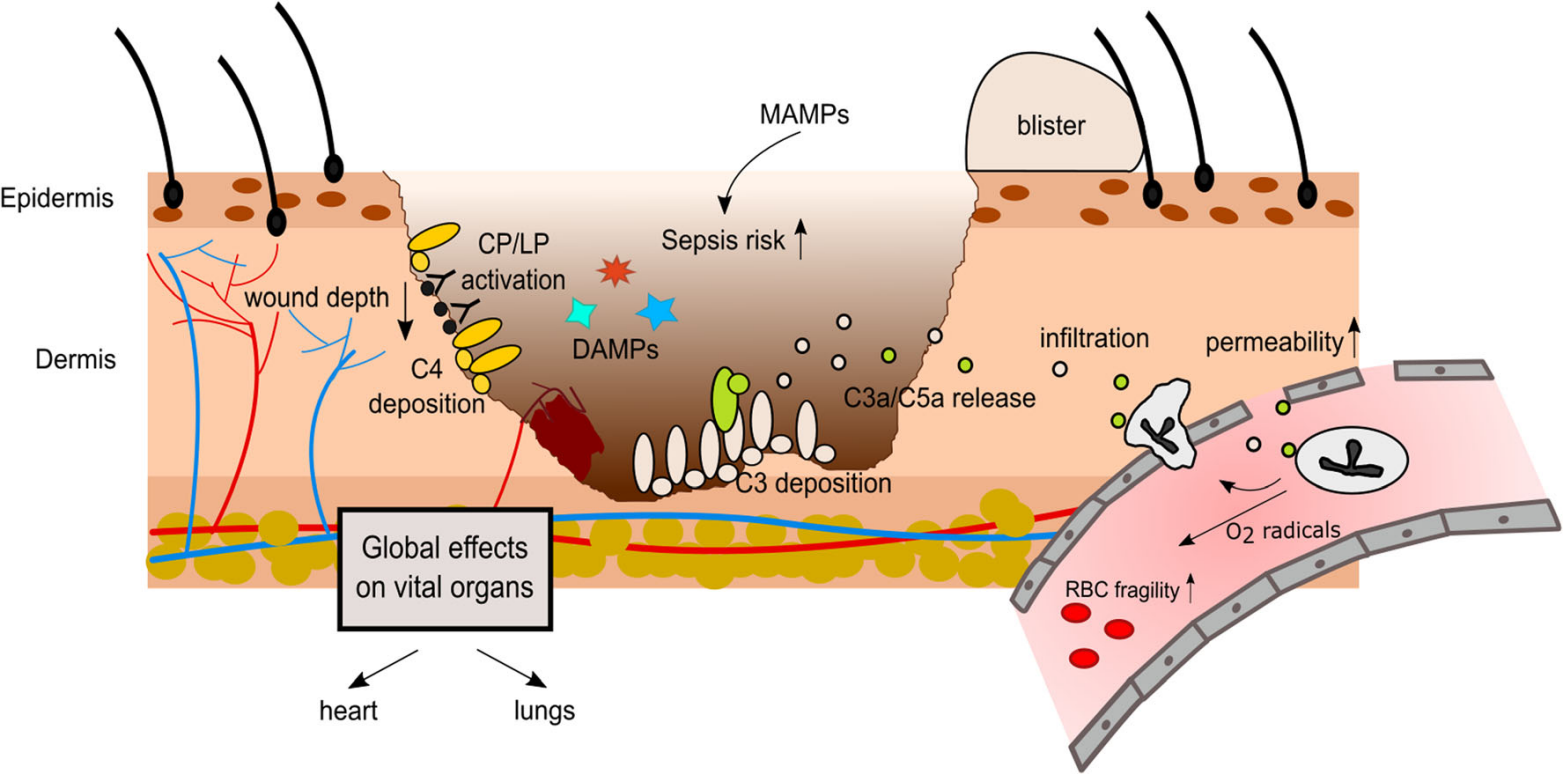
**Complement activation after burn injury**. Burn injury triggers the release of damage associated molecular patterns (DAMPs) and increases the susceptibility for microbial associated molecular pattern (MAMP) exposure, thus elevating the risk for sepsis. Complement activation takes place at the burn site, locally increasing vascular permeability and promoting infiltration of immune cells. Massive anaphylatoxin release also induces widespread effects, especially affecting the lungs and the heart. CP: classical pathway, LP: Lectin pathway, RBC: red blood cell.

Indeed, its involvement has long been known and it sets in rapidly. Sriramarao and DiScipio detected C3 and FH deposition in murine blood vessels in and proximal to the burn site within 10 min of injury [94]. Beside the mechanical leakage from the wound, locally an enhanced vascular permeability was recognized to occur quickly post-burn resulting in an accumulation of water, protein levels, and salt in the interstitium. In a rat model, this effect was shown to be clearly complement dependent since it could be attenuated by prior complement depletion [95]. In addition to these local processes, complement also triggers fast and delayed systemic burden reactions. For instance, intravascular hemolysis of red blood cells or at least an elevated osmotic fragility was recognized early after burn as a consequence of oxygen radicals released from complement-activated neutrophils [96] (see Figure 1). Lung injuries in rats have been displayed to evolve hours after burn in accordance with alterations in complement levels [97, 98]. Complement activation was revealed to occur mainly via the AP evidenced by a massive drop of the hemolytic activity in the alternative pathway (AH50) in the first hours following trauma, while the hemolytic activity of the classical pathway (CH50) remained normal or even supranormal [99]. C3 plasma levels are inversely correlated with the burn severity and could be used as a prognostic marker [100]. However, in the course of 2 weeks after burn, AH50 and CH50 increase concomitant with the presence of complement proteins (e.g., C3 and C4). Having reached a small plateau after 7 days, the further increase of these parameters was statistically more pronounced in survivors compared to non-survivors [101]. Anaphylatoxin plasma concentrations were reported to raise immediately after burn. C3a levels peaked 1 week after burn injury, whereas the literature is not consistent for C5a levels over the first weeks [102, 103]. Remarkably, fluctuations of C3, C3d, and factor Ba concentrations were observed even up to 1 year post-burn, and long-term elevated C3a/C3-ratio baseline levels indicate chronic inflammatory conditions [104, 105]. While a clear relationship between anaphylatoxin concentrations and the development of septic conditions could not be identified [103], elevated C5a plasma levels have been shown to correlate with neutrophil dysfunctions in terms of altered migratory behavior and diminished chemotactic responsiveness to activated serum [102]. Interestingly, complement depletion prior to burn injury by the application of cobra venom factor in a murine burn model lowered mortality rate in the first 24 h post-burn [99]. It is therefore doubtless that complement is a major driver in the pathophysiology of burn not only locally but also systemically (reviewed in [105]). This implicates complement as a potential therapeutic target which is going to be discussed in the following.

Starting from the very top of the complement cascade, application of the C1INH displayed protective effects against ischemic lesions following burn in a porcine model. Therapeutic intervention with C1INH could prevent the damage of subdermal spheres [106]. In comparison to the control group, early-stage complement blockage by C1INH could reduce granulocyte attachment to endothelial cells as well as thrombus formation in the lower dermal vasculature. Moreover, systemic application of C1INH attenuated post-burn shock-induced organ alterations as well as gut-derived bacterial translocation in pigs exposed to a second-degree skin burn [107, 108]. Beside these short-term effects, recently a treatment with C1INH up to 15 days post-burn and its long-term consequences concerning wound healing was investigated [109]. Despite reduction of the local inflammation in the wound, long-term wound healing as measured by necrosis, re-epithelialization, and wound contraction was not affected. Prolonged administration of C1INH also diminished burn-induced infiltration of inflammatory cells (e.g., macrophages) and markers (e.g., C3) not only in the local wound but also in ventricles or atria of the heart [110]. This is of highest importance since burn severity is directly linked to cardiac dysfunctions characterized by tachycardia, myocardial oxygen consumption, and an increased cardiac output which persist for up to 2 years or even longer as measured in children with ≥40% TBSA [111]. Beside elevated levels of catecholamines, which were considered to be the major driver in this study, the engagement of C5a with its receptor on cardiomyocytes was revealed to account for a substantial part in cardiac dysfunctions in other settings [112]. Indeed, C5aR expression increased time-dependently after burn up to 48 h in cardiomyocytes [113]. In a full-thickness scald injury rat model, Hoesel et al. demonstrated benefits of an anti-C5a therapy by significantly attenuating cardiac dysfunction in vivo and also in vitro on isolated cardiomyocytes. LPS treatment of isolated cardiomyocytes disclosed impaired contractility, an effect that could be partially reversed when the rats were pretreated with an anti-C5a inhibitor [113]. Similar protective benefits of an anti-C5a treatment were also shown for lungs in burn-injured rats [114]. Blocking C5a resulted in a significant reduction of ICAM-1 upregulation and caused in turn a reduction in neutrophil accumulation in the lung tissue.

Burn injury is accompanied by an additional local damage caused by the overwhelming acute inflammatory processes. This was attributed to the recognition of self-antigens by natural IgM antibodies and is crucial for the determination of the wound depth [115]. Since this could activate the CP, Suber et al. assayed the wound healing in C4-/- mice in comparison to wildtype mice. While in wildtype mice the burn injury wound healing process was characterized by scarring and hair loss, C4-/- mice healed without scar formation and hair loss and revealed attenuated neutrophil infiltration [115].

Taken together, these observations clearly indicate that the role of complement in burn injury is multidimensional, eliciting effects not only locally but also systemically having tremendous impact on the functionality of several vital organs. Promising results concerning complement inhibition in animal studies have not yet been transferred into human. Moreover, to our knowledge, no studies investigated direct blockage of the central complement proteins, although C5 inhibitors are clinically approved and in case of C3 inhibitors clinically investigated.

## Sepsis

By the past definition of sepsis, a sportsman working out excessively could have easily been characterized as suffering from a systemic inflammatory response syndrome (SIRS) or sepsis. People excising exhaustively feature all signs of a SIRS which is defined by tachycardia, tachypnoea, elevated temperature, and leukocytosis. If the sportsman would have brushed his teeth at the end of the sportif performance, e.g., at the finishing line of a marathon course, he would even temporarily face a bacteremia and thus by definition would be in full-blown sepsis which had been defined as SIRS plus bacteremia [116].

**Figure 2.**
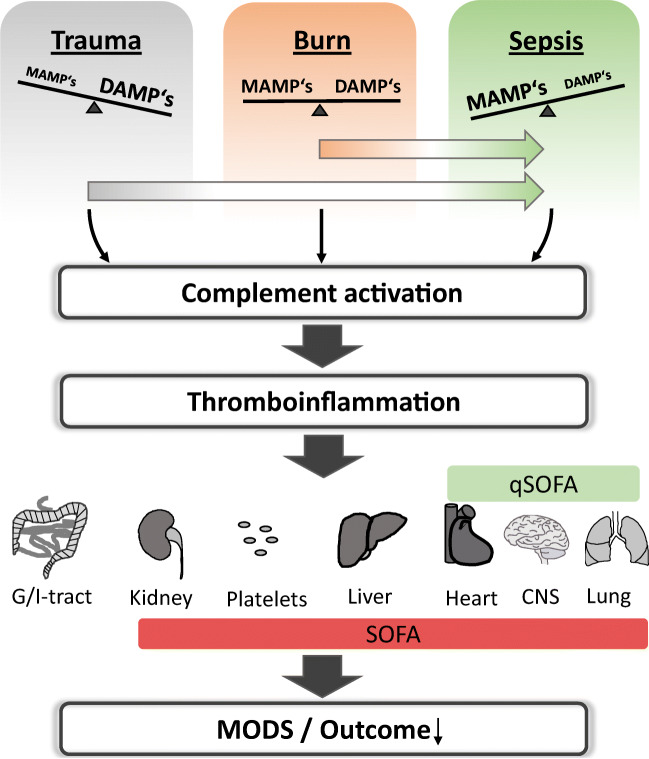
**From acute injury to multi organ dysfunction**. Acute injuries with distinct DAMP/MAMP profile can induce an unbalanced complement activation not only locally but also systemically. A tight linkage to other systems of the innate immune system means that these branches are eventually also activated resulting in a state often called thromboinflammation. The consequences are multidimensional, eliciting effects on various vital organs and worsen the patient‘s outcome. The sequential related organ failure assessment score (SOFA) and especially the quick SOFA (qSOFA, comprising the organs heart, CNS and lungs) provides a basis for the evaluation of a patient‘s septic state. MODS: multi organ dysfunction syndrome.

However, introducing a new definition for sepsis that focuses more on the multi-organ level has solved the dilemma of characterizing exercising athletes as septic patients. In 2016, sepsis has been redefined as a life-threatening organ dysfunction caused by a dysregulated host response to infection [117]. A quick diagnosis of life-threatening sepsis at the bedside can be accomplished by the quick sequential organ failure assessment score (qSOFA). This score focuses on the dysfunction of three vital organs: the brain, heart, and lungs by determination of mental alterations, drop in systemic blood pressure below 100 mmHg, and enhancement of the respiratory rate > 22/min. Applying these new criteria, the proactive sportsman in the example given above will now not any longer be classified to be in full-blown sepsis nor in organ failure. Of note, this new definition resulted in a relatively high sensitivity and specificity for enhanced mortality risk by sepsis [117]. In consequence, translational meaningful research on complement will need to shift from research on systemic parameters of complement activation and actions, e.g., of anaphylatoxins plasma levels, toward a more organ-targeted approach (Figure 2). Thus, against the background of the clinical situation, we will cover the role of complement during sepsis not only on the systemic level but also focus on the three qSOFA-assessing organs: the brain, lungs, and heart.

On a molecular level, complement activation by MAMPs results in generation of the anaphylatoxins C3a and C5a which can induce all classical signs of local inflammation (dolor, rubor, calor, tumor, and functiolesa) and are involved in systemic inflammation as well. Multiple experimental and clinical studies have demonstrated significant systemic complement activation during sepsis [118–120]. In humans, enhanced C3a, C5a, and sC5b-9 concentrations in the blood and corresponding loss of the C5aR1 and C5aR2 have been proposed as driver for sepsis-induced complications and multiple organ failure [120–123]. A recent translational study has shown that C5a could activate a “metabolic switch” in neutrophils as first line of defense resulting in an enhanced intracellular pH and glycolytic flux with mounting of a lactic acidosis in the extracellular microenvironment [74]. Remarkably, lactic acidosis could not only be caused due to an oxygen debt but rather to an activated innate immune response. Furthermore, C5a seems also to form a procoagulant platform on leukocytes by activating a “coagulatory switch” [124]. C5a seems to link the innate and adaptive immune response during the sepsis course as it also induces detrimental effects on natural killer T and natural killer cells [125].

Taken together, complement activation in sepsis is a major driver for the thromboinflammatory response. Besides the systemic effects, complement may act locally, i.e., compartmentalized at various organ levels. The focus in the following section is set on the main organs contributing to the clinical definition of sepsis: the brain, heart and lungs.

## Complement interactions in the brain

The complement system is an integral part of the cerebral innate immune system [126, 127] and can also be recruited from systemic circulation in case of a damaged blood-brain barrier (BBB) [128].

In a murine sepsis model to study the role of complement in brain pathology, LPS injection resulted in an enhanced cerebral expression of glial fibrillary acidic protein (GFAP), toll-like receptor (TLR) 4, pro-inflammatory molecules, Akt-mediated apoptotic events, and increased gliosis. All of these observations were ameliorated in mice overexpressing the murine complement inhibitor, CR1-related y (Crry-tg) [129]. Further downstream, the anaphylatoxin C3a has recently been investigated after LPS injection into neonatal rodents [130]. The authors showed by immunofluorescence enhanced levels of C3a and C3aR in the periventricular white matter of septic animals. It was proposed that LPS can induce generation of C3a and IL-1ß in astrocytes and microglia which will bind to oligodendrocytes and activate the AKT signalling pathway which inhibits the downstream WNT/β-catenin signalling pathway, finally leading to axonal hypomyelination of the periventricular white matter. Remarkably, intraperitoneal application of a C3aR antagonist (SB 290157) reversed all the sepsis-induced alterations of the white matter, indicating C3a as a central player in septic cerebral dysfunction [130]. In contrast, in a murine model of lethal endotoxin shock, C3a-specific expression in the central nervous system (CNS) (without the requirement of previous complement activation) in C3a/GFAP transgenic mice was more protected than C3aR-deficient mice (C3aR-/-) or wildtype littermates [131]. Moreover, C3a/GFAPxC3aR-/- hybrids were also resistant against endotoxin shock, suggesting some beneficial effects of local, cerebral C3a [131]. In this context, C3a has been shown capable to inhibit excitotoxicity-mediated neuronal cell death [132].

In experimental murine sepsis induced by CLP, a considerable blood-brain barrier (BBB) breakdown was found which, of note, was almost completely inhibited by blockade of systemic C5a. Furthermore, sepsis-caused pituitary gland dysfunction was improved by anti-C5a treatment [133]. These findings are supported by a report demonstrating that C5a drives the BBB breakdown in a systemic lupus model [134]. However, some debate remains on whether C5a blockade is beneficial or detrimental for sepsis-associated brain dysfunction [135].

In the clinical setting, sepsis-induced brain dysfunction as determined by altered neuroinflammatory markers, magnetic resonance imaging (which revealed morphological alterations in approximately 2/3 of the patients), and clinical parameters (such as the Glasgow outcome score extended) has shown enhanced C4d but significantly reduced systemic iC3b and C5a concentrations in patients with cerebral lesions [136]. However, the local complement activation level in the brain tissue was not determined. In an experimental animal model setting, this question was investigated previously by this group: in rodent CLP sepsis, significantly enhanced levels of C5a, C5aR, and C3aR were measured in brain samples of septic animals up to 10 days post-infection [137].

An interesting therapeutic approach to improve the energy support has recently been published: mitochondrial transplantation in CLP septic mice improved not only bacterial clearance but also organ performance and survival. A hypothesis-free expression profiling revealed complement and coagulation factors as central contributors to the induced changes [138].

Taken together, complement activation and action in the brain during sepsis seem to alter a delicate balance of pro- and anti-inflammatory effects and requires further mechanistical and clinical research before clinical translation appears feasible.

## Complement interactions in the heart

For more than a quarter of century, complement activation has been postulated to alter the heart function and subsequently the macro- and microcirculation [139]. After exposure of human healthy volunteers to LPS, as expected, the blood pressure dropped, and both, the heart rate and temperature, increased time-dependently. However, application of C1INH failed to alter these hemodynamic alterations although less pro- and more anti-inflammatory mediators were detected systemically, indicating that C1 activation did not alter heart function in this setting [140]. In canines, injected with *Escherichia coli* (*E. coli*), the genetic absence of C3 aggravated the hemodynamic effects with compromised ejection fraction of the left ventricular [141]. In contrast to that, in baboons being exposed to *E. coli*, the reduction in mean systolic arterial pressure (as sepsis sign) was greatly improved among other organ functions by blockade of C3 by compstatin even when applied in a delayed fashion up to 5 h post-infection [142]. Regarding the C5 level, the application of the macrocyclic peptide inhibitor of C5 cleavage (RA101295) improved the hemodynamic readouts [143]. Further mechanistic insights of the interaction between complement activation products and the heart were obtained in vivo in rodent sepsis models and in vitro with cardiomyocytes [112]. The mosaic picture of these studies suggests that systemic C5a interacts with the C5aR1 and C5aR2 on cardiomyocytes [144, 145] alters the membrano-electrophysiological features [146] and induces intracellular signalling [147]. In turn, key calcium-regulatory proteins (such as sarco-/endoplasmic reticulum Ca2+-ATPase), transporters, and channels become dysfunctional all of which lead to an intracellular calcium disbalance and contractory dysfunction [112, 146]. In association with released histones (presumably from activated leukocytes), C5a can activate the NLRP3 inflammasome in cardiomyocytes and contribute to the local cardio-suppressive inflammatory milieu [148, 149] [150]. Of note, blockade of the C5a-C5aR interaction maintained the C5aR expression and hemodynamic performance of the heart [112, 146].

On the MAMP sensing site, TLRs are mainly involved in mediating inflammation and organ injury during sepsis. Concerning the heart, TLR signalling is involved in sepsis-induced functional suppression [151]. Activation of TLR2, TLR3, and TLR4 resulted in robust expression of complement FB by cardiomyocytes. In murine CLP sepsis, FB was indeed enhanced in the heart and associated with cardiac deposition of C3 fragments [152]. Moreover, absence of FB resulted in some protective cardiac function and improved the outcome. The absence of MyD88 as common adaptor protein of most TLRs attenuated the upregulation of FB and C3 in cardiomyocytes [152]. These findings support a combined inhibition approach of both, the TLR and complement activation axis. Indeed, blockade of CD14 as co-receptor along with TLR4 for LPS and synchronic inhibition of C5 in a pig sepsis model revealed some protective effects on the generation of cytokines from cardiac cells [153]. However, further clinically meaningful functional hemodynamic data need to be established. In addition, the interplay between adrenoreceptors as drivers of the heart performance and complement receptors, such as the C1 receptor and C5aR1, needs further mechanistic elucidation [154].

Taken together, broad evidence indicates that C3 and C5 activation is central in development of cardiac dysfunction during sepsis, and it is tempting to speculate that blockade of these activation products will improve the cardiac performance as key component of septic complications also in translational settings.

## Complement interactions in the lungs

The lungs are main actors and targets of the immune-pathophysiological response during sepsis and therefore a site of massive complement activation. Pulmonary thromboinflammation, in principle investigated more than two decades ago [155, 156], is in renaissance due to the current SARS-CoV-2–induced pulmonary pathology. Systemic and pulmonary complement, especially C3 and C5 activation, has been proposed as reasonable therapeutic targets which is under ongoing clinical assessment [157–159].

Multiple experimental data sets were collected in animal models of sepsis demonstrating morphological and respiratory changes [160]. Accordingly, the following principal sequelae can occur as briefly outlined: MAMP-induced complement activation leads to C3a and C5a liberation, which upregulates expression of pulmonary endothelial adhesion molecules (e.g., selectins) and thereby recruits neutrophils to the alveolar space. Upon activation (again by anaphylatoxins), these first responder cells release more inflammatory chemo- and cytokines and thereby recruit more inflammatory cells. Clearance of bacteria is accomplished by cellular activation and subsequent release of proteases and mounting of an oxidative burst, all of which also damage host tissue and the air-blood barrier. The resulting pulmonary inflammation and protein/water leakage leads to difficulties in oxygen and carbon dioxide diffusion which increasingly generate an oxygenation problem, clinically manifested as respiratory dysfunction or even failure.

Concerning deployment of complement therapeutics during experimental sepsis, complement depletion [161], C1 inhibition [162], specific blockade of C5a [118, 163], C5aR1 [164], genetic deficiency in C5 [165], or genetic absence of C6 [166, 167] have been demonstrated to improve sepsis-induced pulmonary inflammation on a morphological and/or functional level.

In contrast, in the case of C3, two independent reports in murine sepsis models suggested that application of C3 will support pulmonary clearance and improve respiratory function and outcome during sepsis [141, 168]. In line with this, genetic absence of C3 leads to signs of pulmonary injury and multiple organ injury, and general outcome during sepsis is much worse than in wildtype littermates indicating overall protective effects of C3 in case of early bacterial sepsis [169, 170].

In clinical sepsis, it is established that patients reveal enhanced systemic concentrations of complement activation products such as C3a and C5a with inconclusive reports concerning their reliability to predict development of pulmonary dysfunction, clinically manifested as acute lung injury (ALI) and acute respiratory distress syndrome (ARDS) [120, 171–173]. Enhancement of pulmonary alveolar-capillary permeability has been determined in patients after trauma and with sepsis who were at risk to develop ARDS. Elevated plasma levels of C3a_desarg_ correlated somehow with the air-blood barrier leakage [173]. In line, deposition of immune complexes along with C3 has been suggested in one human autopsy study as a pathomechanism of sepsis-induced multiple organ failure including the lungs [174]. Similar findings were reported in septic patients suggesting that both the classical and alternative pathways are activated before the onset of ARDS [175]. This was evidenced by the enhanced systemic appearance of the C1rC1s-C1 INH complex (derived from CP activation) and C3b-properdin complex (derived from AP activation) concomitant with the sC5b-9 2 days prior to ARDS [175].

Regarding therapeutic strategies, the application of anti-C5a_desarg_ antibodies in non-human primates challenged with *E. coli* resulted in significantly improved oxygenation and decreased extravascular lung water in comparison to the non-treated septic group and, thus, attenuated septic ARDS [176]. In the same model, septic monkeys showed a rapid activation of systemic complement (with depletion of C3 and C5 and generation of C3a and C5a) which was accompanied by a significant fall in peripheral leukocyte count and a significant increase in the pulmonary sequestration of leukocytes 15 min post-infection [177, 178]. In baboons with *E. coli* sepsis-induced ALI, blockade of C3 by compstatin significantly improved the acute alveolar inflammation as confirmed by diminished micro thrombosis, leukocyte infiltration, and capillary leakage. Of note, anti-C3 treatment also influenced extracellular matrix remodelling by attenuating collagen deposition and the development of fibrosis [142, 179].

Studies on therapeutic interventions in humans with septic respiratory failure are rare. A previous study on seven mechanically ventilated patients with streptococcal toxic shock, who were treated with C1INH, revealed a significant improvement of the capillary leakage syndrome in all but one patient [180]. One recent study applied a C5a antibody (InflaRx) to septic patients but did not report significant improvement on the respiratory function. However, since the clinical development of complement therapeutics in the respiratory field may become boosted by the current SARS-CoV-2 pandemic, further deployment in the field of sepsis is to be expected.

## Conclusion

The complement cascade is the major effector arm of the molecular innate immune system and interacts, involving multiple of its activation products, with several other branches of the immune system as well as the key regulatory circuits which serve tissue homeostasis and hemostasis. As the complement system constantly probes self, altered, and foreign surfaces for their potential to inflict harm, it comes as no surprise that substantial or even exuberant complement activation is a consequence of several traumatic injuries like trauma, burn, and sepsis. All these conditions are characterized by massive tissue damage which triggers strong complement activation. Thus, complement activation in these conditions usually exceeds the checkpoints that separate the non-inflammatory proximal complement cascade producing C3 activation products from the inflammatory terminal pathway which liberates the potent anaphylatoxin C5a and initiates MAC formation. In vivo evidence from animal studies and clinic settings demonstrates that exuberant complement activation in these conditions participates in causing systemic inflammation ultimately leading to organ failure. However, knowledge on what complement pathways or activation products are exactly driving detrimental inflammation leading to organ dysfunction is still sparse. Progress at this end is hampered by inconsistent findings reported in the literature which are based on different animal models and the (so far) rather sparsely available clinical studies investigating in humans. A further complicating factor in delineating how complement activation could be best intercepted therapeutically in these conditions is that local activation in tissues has been described to drive other physiological pathways than systemic complement activation. Clearly more research is necessary to further our understanding when, where, and for how long complement is best inhibited for alleviating morbidity and mortality in these complex clinical conditions. Cumulating evidence indicates that the terminal pathway (or specific parts thereof—e.g., anaphylatoxin C5a) is one of the most promising/best documented complement pathways to be interfered with in future translational studies. However, in some disease models, inhibition of proximal complement pathways has resulted in remarkable benefits calling for further studies that dissected the role of the many different complement activation products upstream of C5 activation (e.g., C3a, C3b, iC3b, C3dg, C4a, C4b, iC4b) in trauma, burn, and sepsis.

## Abbreviations

AH50: Hemolytic complement activity of the alternative pathway
ALI: Acute lung injury
AP: Alternative pathway
ARDS: Acute respiratory distress syndrome
ATP: Adenosine triphosphate
BBB: Blood-brain barrier
C1INH: C1 inhibitor
C4BP: C4b-binding protein
CH50: Hemolytic complement activity of the classical pathway
CLP: Cecal ligation and puncture
CNS: Central nervous system
CR1: Complement receptor 1 (CD35)
CRP: C-reactive protein
Crry-tg: Complement receptor 1–related gene/protein y
CP: Classical pathway
Cp40: C3 peptide inhibitor of the compstatin family
DAF: Decay acceleration factor (CD55)
DAMP: Damage-associated molecular pattern
DNA: Deoxyribonucleic acid
E. coli: *Escherichia coli*
FB: Factor B
FH: Factor H
FHL-1: Factor H-like 1
FHR: Factor H related
FI: Factor I
GFAP: Glial fibrillary acidic protein
Ig: Immunoglobulin
IIIS: Intravascular innate immune system
IRI: Ischemia-reperfusion injury
LP: Lectin pathway
LPS: Lipopolysaccharide
MAC: Membrane attack complex
MAMP: Microbe-associated molecular pattern
MAP: Middle artery pressure
MASP: Mannan-binding lectin-associated serine protease
MCP: Membrane co-factor protein (CD46)
MCP-1: Monocyte chemotactic protein 1
mmHg: Millimeter of mercury
MyD88: Myeloid differentiation factor 88
qSOFA: Quick sequential related organ failure assessment score
sC5b-9: Soluble C5b-9 complex
SIRS: Systemic inflammatory response syndrome
TBSA: Total body surface area
TCC: Terminal complement complex
TLR: Toll-like receptor
